# Vascular retinal findings after coronavirus disease 2019 vaccination: a correspondence

**DOI:** 10.5935/0004-2749.2022-0178

**Published:** 2025-08-21

**Authors:** Beuy Joob, Pathum Sookaromdee, Viroj Wiwanitkit

**Affiliations:** 1 Sanitation Academic Center, Bangkok, Thailand; 2 Private Academic Consultant, Bangkok, Thailand; 3 Dr DY Patil University, Pune, India; 4 Joseph Ayobabalola University, Ikeji-Arakeji, Nigeria

Dear Editor,

We would like to share our views on the article “Vascular retinal findings after COVID-19 vaccination in 11 cases: A coincidence or consequence?^(1)^.” Retinal incidents linked to COVID-19 immunization are conceivable, but uncommon, according to Silva et al. The association between these events and post-COVID-19 immunization warrants detailed investigation before a meaningful conclusion can be drawn^(1)^. The vascular retinal disease can develop after immunization, and it could be an outcome or a coincidence. Therefore, the differential diagnosis is necessary, although it must be derived after a thorough understanding of the problem. When a COVID-19 vaccination recipient complains of poor vision, the treating physician must conduct a thorough anamnesis and physical examination to determine the underlying cause^(2-3)^. Investigating the use of retinal imaging can be beneficial in such cases. However, any conceivable cause of renal vascular disease must be eliminated before confirming the connection. The unfavorable effect of the COVID-19 vaccine may be overlooked for a thorough examination when mass vaccination is required. The precise data from verification can be crucial in reducing apprehension toward vaccination.
